# Anatomy of the Ediacaran rangeomorph *Charnia masoni*

**DOI:** 10.1002/spp2.1234

**Published:** 2018-09-11

**Authors:** Frances S. Dunn, Philip R. Wilby, Charlotte G. Kenchington, Dmitriy V. Grazhdankin, Philip C. J. Donoghue, Alexander G. Liu

**Affiliations:** ^1^ School of Earth Sciences University of Bristol Life Sciences Building, Tyndall Avenue Bristol BS8 1TQ UK; ^2^ British Geological Survey Nicker Hill, Keyworth Nottingham NG12 5GG UK; ^3^ Department of Earth Sciences Memorial University of Newfoundland St John's NL A1B 3X5 Canada; ^4^ Trofimuk Institute of Petroleum Geology & Geophysics Prospekt Akademika Koptyuga 3 Novosibirsk 630090 Russia; ^5^ Novosibirsk State University Pirogova Street 1 Novosibirsk 630090 Russia; ^6^ Department of Earth Sciences University of Cambridge Downing Street Cambridge CB2 3EQ UK

**Keywords:** Ediacaran, rangeomorph, morphology, intraspecific variation, taxonomy

## Abstract

The Ediacaran macrofossil *Charnia masoni* Ford is perhaps the most iconic member of the Rangeomorpha: a group of seemingly sessile, frondose organisms that dominates late Ediacaran benthic, deep‐marine fossil assemblages. Despite *C. masoni* exhibiting broad palaeogeographical and stratigraphical ranges, there have been few morphological studies that consider the variation observed among populations of specimens derived from multiple global localities. We present an analysis of *C. masoni* that evaluates specimens from the UK, Canada and Russia, representing the largest morphological study of this taxon to date. We describe substantial morphological variation within *C. masoni* and present a new morphological model for this species that has significant implications both for interpretation of rangeomorph architecture, and potentially for existing taxonomic schemes. Previous reconstructions of *Charnia* include assumptions regarding the presence of structures seen in other rangeomorphs (e.g. an internal stalk) and of homogeneity in higher order branch morphology; observations that are not borne out by our investigations. We describe variation in the morphology of third and fourth order branches, as well as variation in gross structure near the base of the frond. The diagnosis of *Charnia masoni* is emended to take account of these new features. These findings highlight the need for large‐scale analyses of rangeomorph morphology in order to better understand the biology of this long‐enigmatic group.

The emergence of animals is among the most formative evolutionary events in Earth history, yet our understanding of early animal evolution remains poorly constrained. Molecular estimates place the origin of Metazoa somewhere between 700 and 800 million years ago (dos Reis *et al*. 2015) but few body fossils of undisputed animal affinity are known from strata older than latest Neoproterozoic (e.g. Cunningham *et al*. 2017). Some of the best candidates for pre‐Cambrian animals are members of the Ediacaran macrobiota: a disparate group of largely soft‐bodied macroscopic organisms that lived in marine environments during the final *c*. 30 million years of the Ediacaran Period (Grazhdankin 2014; Budd & Jensen 2017). Despite the potential significance of these fossils for understanding early animal evolution, only a small number of Ediacaran macrofossil taxa have been morphologically well‐characterized following study of large populations of individuals (e.g. Vickers‐Rich *et al*. 2013; Evans *et al*. 2017; Hoekzema *et al*. 2017; Kenchington & Wilby 2017). Typical preservation of the Ediacaran macrobiota (as cast and mould impressions) means that there is uncertainty as to how much of their anatomy is captured, with internal features being particularly rare (though see Dzik & Ivantsov 2002; Narbonne *et al*. 2009; Vickers‐Rich *et al*. 2013). Consequently, most previous suggestions of metazoan affinity for Ediacaran macrofossil taxa are equivocal and based on palaeoecological or developmental evidence in addition to the limited amount of direct morphological information currently available.

The earliest known palaeocommunities of the Ediacaran macrobiota date to *c*. 571–560 Ma (Noble *et al*. 2015; Pu *et al*. 2016) and are found among sedimentary rocks deposited in deep marine palaeoenvironments (e.g. Wilby *et al*. 2011; Liu *et al*. 2012). They are dominated by organisms with a frondose body plan that could reach up to two metres in length (Narbonne & Gehling 2003; Liu *et al*. 2015). Some of these fronds exhibit self‐similar (sometimes considered ‘fractal’) branching and have been assigned to the morphogroup Rangeomorpha (Pflug 1972; Jenkins 1985; Narbonne 2004; Erwin *et al*. 2011), which may comprise a clade (Dececchi *et al*. 2017). The constructional architecture of rangeomorphs has proven difficult to reconcile with the body plans of extant taxa, resulting in multiple competing hypotheses, including both metazoan and non‐metazoan affinities, for members of the group. These interpretations have included algae (Ford 1958), fungi (Peterson *et al*. 2003), lichens (Retallack 1994), total‐group metazoan (Budd & Jensen 2017) and pennatulacean cnidarians (Glaessner 1959). Recent reassessment of developmental data derived from rangeomorphs concluded that most of these interpretations are not compatible with morphogenetic evidence and that rangeomorphs are likely to fall within the total group Metazoa (Dunn *et al*. 2018a).

Recent field and museum visits in Newfoundland (Canada), Charnwood Forest (UK) and the White Sea (Russia) have unearthed new material that includes rangeomorph specimens of markedly different sizes within individual species. Such specimens are interpreted as different developmental stages of the organisms (Liu *et al*. 2012; Wilby *et al*. 2015; Dunn *et al*. 2018a) that provide new opportunities to obtain insight into both rangeomorph anatomy and morphogenesis. The prominent rangeomorph taxon *Charnia masoni* (Ford 1958; Fig. 1A) has a long history of research, broad spatial and stratigraphical distributions and both shallow‐ and deep‐marine environmental tolerance (Grazhdankin *et al*. 2008; Gehling & Droser 2013; Liu *et al*. 2015). New populations of *C. masoni* offer excellent opportunities to test claims of animal ancestry in Ediacaran rangeomorphs.

**Figure 1:**
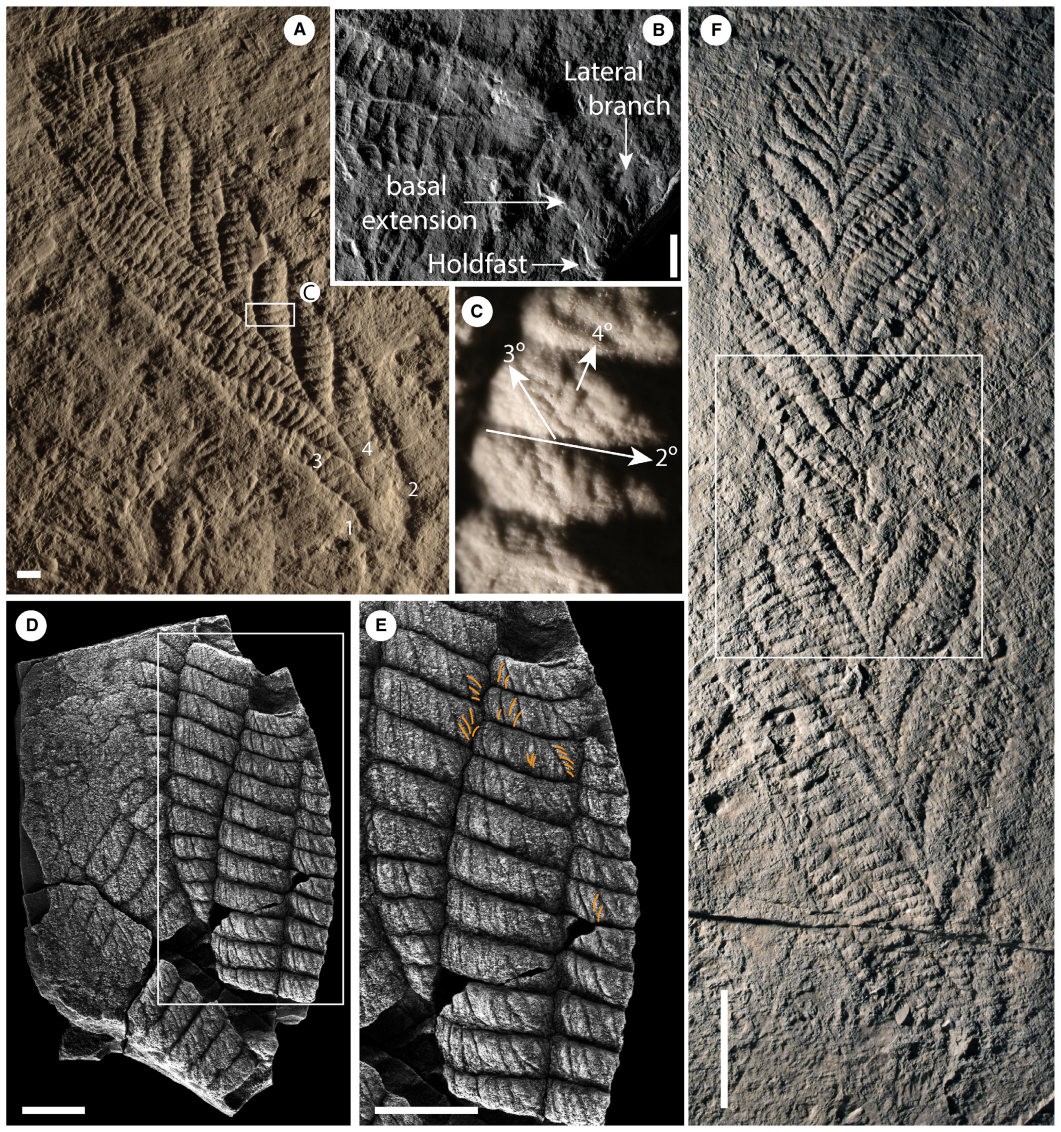
A–C, *Charnia masoni* Holotype (LEIUG 2328) from Bed B (Wilby *et al*. 2011), North Quarry, Charnwood Forest, UK: A, latex mould of the complete specimen; lateral branches (the basal‐most branch pair) are labelled 1 and 2, branches comprising the basal extension (the next most basal branch pair) are labelled 3 and 4; see Dunn *et al*. (2018b) for a reflectance transformation image of holotype specimen; B, cast of the basal region of the holotype, showing the holdfast, basal extension and lateral branches; C, displayed branch architecture in third and fourth order branches (2°, second order branch; 3°, third order branch; 4°, fourth order branch); holotype mould. D–E, partial *Charnia masoni* specimen from the White Sea (PIN 3993‐7018): E, high order rangeomorph branching, examples of rotated or displayed furled fourth order branches are highlighted in orange; F, latex mould of a *Charnia* specimen interpreted as being twisted by Wilby *et al*. (2015) (BGS GSM 105873); the white box highlights the area of inferred twisting. Scale bars represent: 10 mm (A–E); 5 cm (F).

We here present a reanalysis of the morphology of *Charnia masoni* and identify features that lead us to propose a new model for its anatomy. This model has significant implications for our understanding of rangeomorph intra‐specific variation, and consequently for rangeomorph taxonomic schemes. The following redescription is undertaken in the expectation that a detailed understanding of anatomy must necessarily precede understanding of an organism's place in phylogeny and, consequently, its evolutionary significance.

## Previous work

*Charnia masoni* is a uniterminal rangeomorph (see Dunn *et al*. 2018a *a*), which is known to range in length from *c*. 1 to 66 cm (Fig. 1; Boynton & Ford 1995; Hofmann *et al*. 2008; Liu *et al*. 2012). It comprises a holdfast, stem and tapering ovate to parallel‐sided frond (Laflamme *et al*. 2007) consisting of two rows of first order branches (Fig. 1A; terminology follows Brasier *et al*. 2012). First order branches are longest in the middle of the frond and shortest at the distal tip (Ford 1958). *C. masoni* is considered to belong to the Charniida (Pflug 1970; Glaessner 1979); a sub‐group of Rangeomorpha comprising those taxa with single‐sided (rotated; Brasier *et al*. 2012) first order branches (Narbonne *et al*. 2009). The angle of repose of *Charnia* first order branches varies amongst specimens (both within and between bedding planes) but the form of the organism remains constrained (Dunn *et al*. 2018a). First order branches meet in an alternating arrangement at the midline to form a zigzag apico‐basal axis, with no visible stalk (Ford 1958; Grazhdankin 2004a) and, as such, the growth axis has been considered concealed (Brasier *et al*. 2012). This branch alternation confers glide reflection symmetry (an offset form of bilateral symmetry; e.g. Brasier *et al*. 2012) on the frond. Rarely, groups of first order branches may dislocate from their neighbours (Wilby *et al*. 2015, figs 5–10) but more commonly they present as a tightly stacked arrangement.

**Figure 2:**
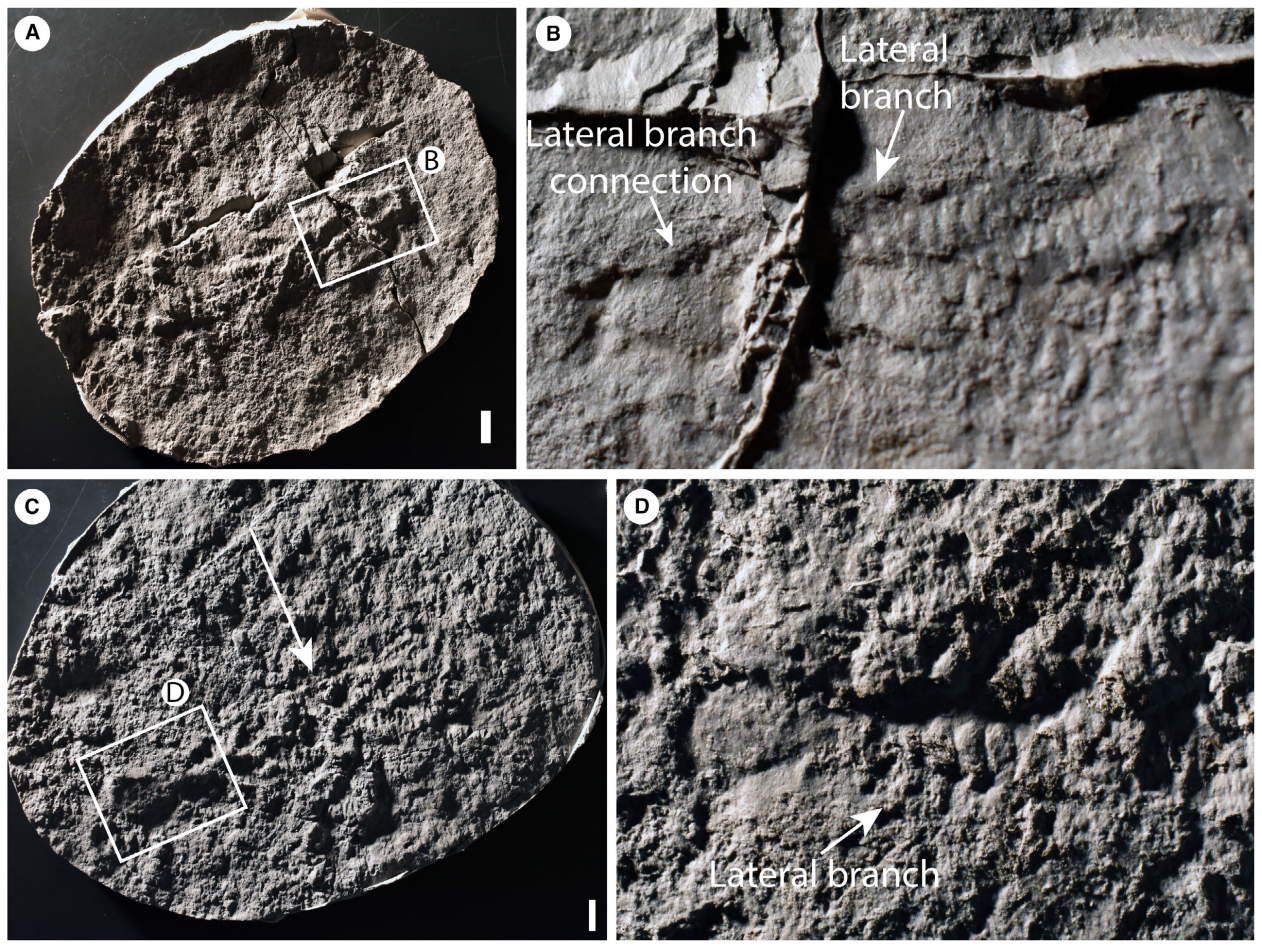
*Charnia masoni* specimens from Charnwood Forest, UK. A–B, cast of BGS GSM 105993; the arrows in B highlight the basal‐most branch as it connects directly to the lateral margin of the holdfast. C–D, cast of BGS GSM 105972; the specimen is arrowed in C; in D, the arrow points to the basal‐most branch, which connects directly to the lateral margin of the holdfast. Scale bars represent 10 mm. Colour online.

First order branches have been described to comprise up to 25 second order branches (Wilby *et al*. 2015), the shape of which may vary from rectangular to sigmoidal along an individual first order branch (Laflamme *et al*. 2007). Second order branches themselves comprise smaller, third (Jenkins 1985) and fourth order branches (Brasier & Antcliffe 2009), with each successive branch order oriented broadly perpendicular to the previous one. The branching in *Charnia masoni* has been described as undisplayed and furled at all orders (*sensu* Brasier *et al*. 2012), with the number of first order branches generally increasing with specimen size (e.g. Antcliffe & Brasier 2008). These observations have led researchers to conclude that *C. masoni* differentiated new first order branches during its life (*sensu* Dunn *et al*. 2018a) and that these branches subsequently inflated as the organism grew further (Antcliffe & Brasier 2007, 2008; Wilby *et al*. 2015). New branches have typically been interpreted to differentiate from the apex of the organism (Antcliffe & Brasier 2007), where the smallest first order branches are located, but an additional basal growth zone has been proposed following identification of stems of markedly different relative lengths in some specimens (Dunn *et al*. 2018a *a*, figs 1A–B, 2E). Whether all four orders of branch division are visible at all observed stages of ontogeny, or whether they emerge during development in a hierarchical fashion (as suggested by Flude & Narbonne 2008), has not yet been resolved.

Although the gross morphology of *Charnia masoni* has been relatively well‐characterized, discrepancies exist in the detail to which its component parts have been studied. The morphology of first order branches has been well analysed (e.g. Laflamme *et al*. 2007; Wilby *et al*. 2015), while third order and fourth order branches have been little discussed in the literature, presumably due to their small size and incomplete preservation within most specimens. There is therefore ample scope for morphological analysis of these smallest branch divisions using well preserved specimens.

*Charnia masoni* is widely considered to have been identical on both faces/sides. However, Grazhdankin (2004a) suggested that this may not have been the case and that one face of *C. masoni* possessed characteristic furled and rotated rangeomorph branching architecture at multiple branch orders, while the other possessed first and second order branches only. Narbonne *et al*. (2009) described putative internal anatomy in one specimen termed ‘*Charnia* cf. *C. masoni*’, identifying a possible central stalk with ‘tube’‐like support structures for the first order and second order branches. They also describe an outer ‘distal rim’ to the frond, which they considered was an internal feature that originally connected to the central stalk and the first order branch support structures (though see Grazhdankin & Seilacher 2005, who interpreted ‘internal’ structures as resulting from current winnowing, or Brasier *et al*. 2013, who reinterpreted both the distal‐rim and the internal stalk as sedimentary features related to scouring).

The holdfast of the organism has received little discussion (though see Jenkins 1985; Grazhdankin 2014), possibly because much work has focused on the holotype specimen, in which the holdfast has historically been thought to be missing (though see Wilby *et al*. 2015 fig. 5‐1). Where present, the holdfast is small and bulbous (Laflamme *et al*. 2007; Wilby *et al*. 2015), though it was described as elongate by Jenkins (1985) and recent work has also suggested that the holdfast of *Charnia masoni* may be more deeply buried than other rangeomorph holdfasts, thus only appearing to be smaller (Burzynski & Narbonne 2015). In a few specimens, a stem‐like region (Dunn *et al*. 2018a *a*), sometimes with second order subdivisions (Wilby *et al*. 2015), can be seen in *C. masoni* connecting the holdfast to the frond (the basal extension as defined here). This region is considered distinct from the true, naked stems of other rangeomorphs (Laflamme *et al*. 2012) and non‐rangeomorph frondose Ediacaran taxa (e.g. Laflamme *et al*. 2004), which do not possess any second order subdivisions along their stems.

In summary, while *Charnia masoni* is one of the best studied rangeomorph taxa, there remain several crucial aspects of anatomy that are either contentious (e.g. internal anatomical structures), or insufficiently characterized. Some of these are features (e.g. branching architecture) that contribute significantly to taxonomic diagnosis in rangeomorphs (Laflamme & Narbonne 2008; Brasier *et al*. 2012). Any improvement to our knowledge of the anatomy of *Charnia* is therefore valuable.

## Material and method

A total of 47 well preserved *Charnia masoni* specimens from Bed B of North Quarry in the Bradgate Formation, Charnian Supergroup, UK (see Wilby *et al*. 2011), including the holotype (LEIUG 2328, Ford 1958), and 17 specimens from Bed LC6 of the Catalina Member of the Trepassey Formation, Newfoundland (see Liu 2016), were studied either in the field or from high resolution casts and moulds (figured specimens are housed at the British Geological Survey, Keyworth and the Sedgwick Museum, Cambridge, respectively). Specimens are preserved in low negative epirelief and occupied deep‐water turbiditic depositional settings during life (Wood *et al*. 2003). Five additional partial specimens from the Verkhovka Formation, Valdai Group, White Sea region of Russia (Grazhdankin 2004a), were analysed from photographs, or at the Paleontological Institute (PIN) in Moscow. These Russian specimens are preserved in three dimensions in fine‐grained sandstone interbeds alternating with mudstone and representing a storm‐influenced middle shoreface depositional environment (Grazhdankin 2004a).

Specimens of *Charnia masoni* from Newfoundland were retrodeformed prior to study (a technique used to account for tectonic deformation of specimens; Wood *et al*. 2003) following the constant area method (Heywood 1933), while specimens from Charnwood Forest were not retrodeformed since all fronds on Bed B are aligned and are considered to have been subjected to the same magnitude of deformation (following Wilby *et al*. 2015). Specimens from the White Sea were not retrodeformed, as the strata are not considered to have undergone significant tectonic deformation (Stankovsky *et al*. 1990; Grazhdankin 2003, 2004b). Due to inherent deformational differences, we do not consider quantitative data derived from these various populations to be directly comparable. However, we do discuss general morphological variation across the different sample areas.

Interpretive illustrations of individual specimens were produced in Adobe Photoshop CC. Silicone moulds were made of specimens from Newfoundland in the field, under permits issued by the Government of Newfoundland and Labrador, under Regulation 67/11 of the Historic Resources Act.

###### Institutional abbreviations

BGS, British Geological Survey, Keyworth, UK; CAMSM, Sedgwick Museum, University of Cambridge, UK; LEIUG, Department of Geology, University of Leicester, UK; OUMNH, Oxford Museum of Natural History, Oxford, UK; PIN, Paleontological Institute, Russian Academy of Sciences, Moscow, Russia.

## Results

### Specimens from Charnwood

The best‐preserved and largest specimens of *Charnia masoni* exhibit four (resolvable) orders of branching (Fig. 1), while the smallest specimens (*c*. 2 cm) lack the resolution required to determine the number of branch orders originally present. The smallest first order branches are located at the distal tip of individual fronds, which are typically ovate in shape and appear well constrained (i.e. lacking first order branches of aberrant length) in all specimens. One specimen appears to show an area of first order branch dislocation (*sensu* Wilby *et al*. 2015) with the angle of repose of first order branches being higher above the dislocated area (towards the distal tip; Fig. 1F). First order branches are constructed of rectangular second order branches, which are oriented laterally and basally and are themselves constructed of third and fourth order branches. Third order branches, which are oriented apically, can appear displayed and furled (Fig. 1C, terminology *sensu* Brasier *et al*. 2012), undivided, or rotated and furled.

**Figure 3:**
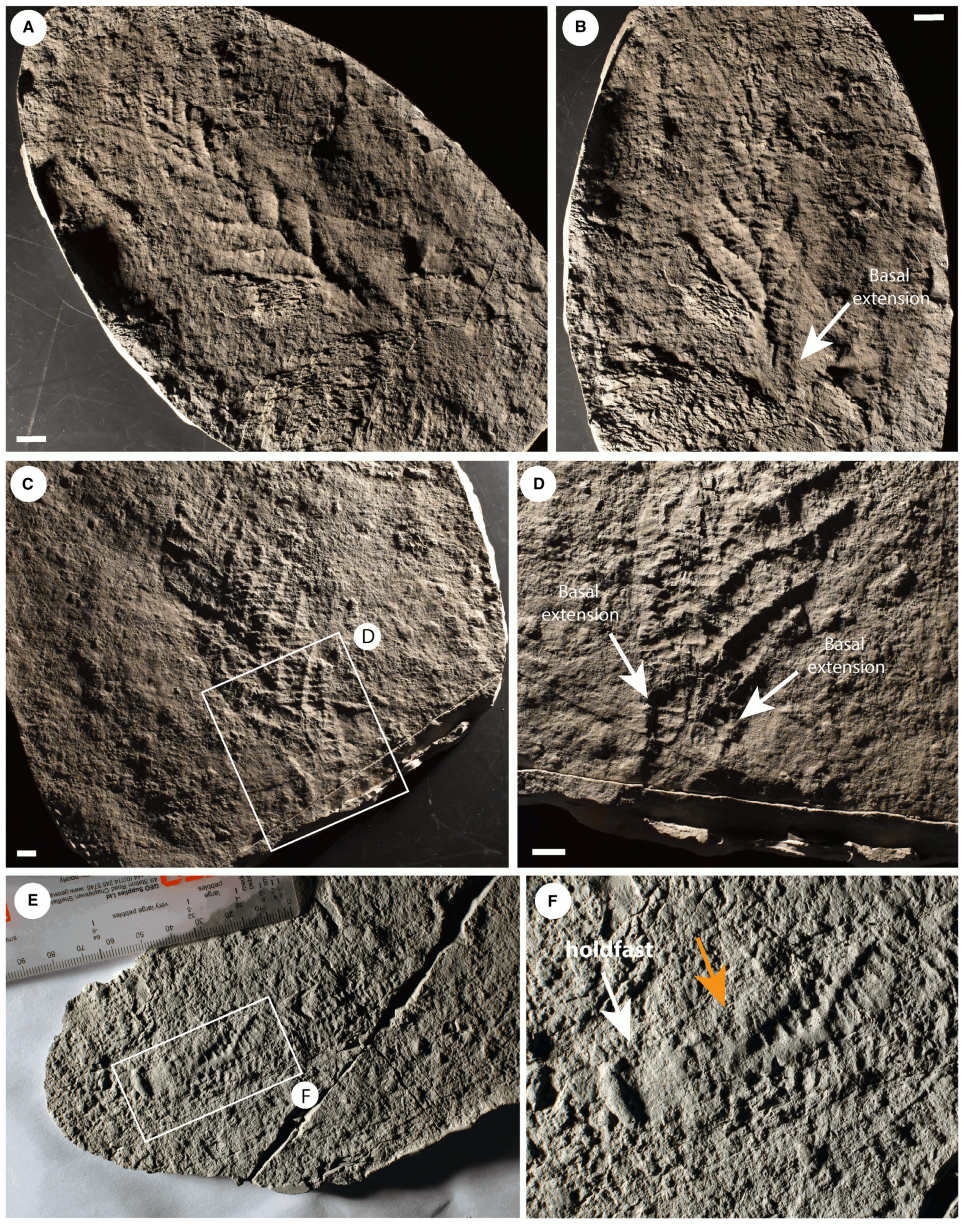
*Charnia masoni* specimens from Charnwood Forest, UK. A–B, cast of BGS GSM 106078, showing the basal extension. C–D, cast of BGS GSM 105997, showing the basal extension in D; this specimen does not preserve a holdfast. E–F, cast of BGS GSM 105966, which does not show a basal extension, but rather the first order branches connect to the holdfast without any expansion near the base of the branch; the holdfast and lowermost branches are arrowed in F (left and right arrows respectively). Scale bars represent 10 mm. Colour online.

**Figure 4:**
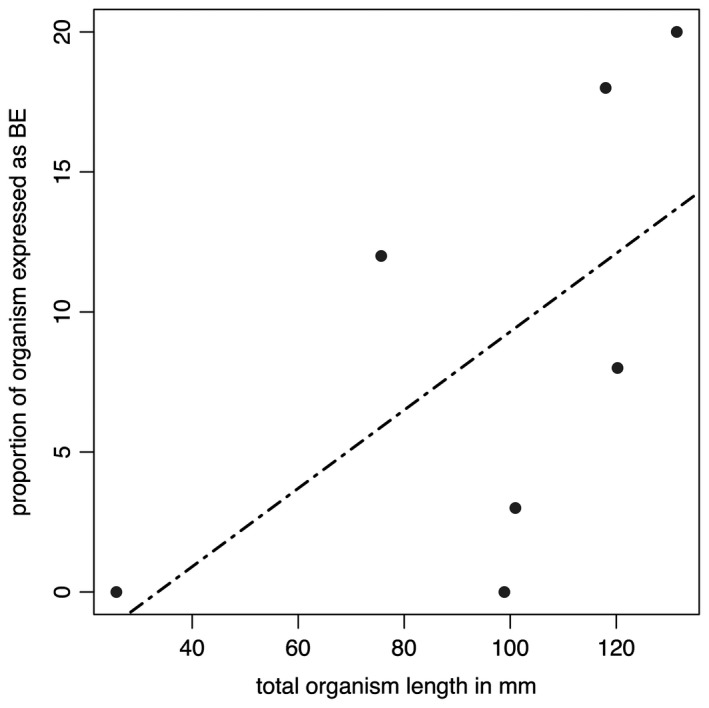
Data from Table 1 plotted in graphical form. The black dashed line represents the best fitting (linear) model (AICc = 59.12972), but this is non‐significant (*p* = 0.1483).

Most first order branches appear to meet in an alternating arrangement in the centre of the organism, conferring a glide symmetrical arrangement. However, the two most proximal branches in individual specimens (closest to the holdfast) do not appear to conform to this pattern, instead connecting directly to the lateral margins of the holdfast (Figs 1A–B, 2). These two most proximal branches (observed to be present in eight specimens and absent from eight specimens, based on the position of their unique attachment point) are distinct from all other first order branches, with second order branches present along their entire length and third order branches sporadically preserved. We term this pair of first order branches the lateral branches. The next most‐apical pair of first order branches (i.e. the second pair of first‐order branches; Figs 1A–B, 3) may also appear morphologically distinct, in some cases extending between the most proximal first order branch pair (the lateral branches) to form an area previously termed the ‘stem’ or stem‐like area (Dunn *et al*. 2018a *a*). This area is variable among specimens; it can be present or absent within individuals from a single population (it is present in 9 specimens from Charnwood Forest, out of 19 where the base of the organism is preserved) and it may vary in length within the population (both in absolute and proportional terms; see Fig. 4, Table 1).

**Table 1:** Measurements of total specimen length, the length of the basal extension and the relative proportion of the specimen this area comprises

Specimen	Total length (mm)	Basal extension (mm)	Length of the basal extension as a proportion of total organism length (%)
GSM 105978	118	20.84	18
GSM 106040	>111.31	11.61	N/A
GSM 105966	98.89	0	0
Holotype	>220.09	26.61	N/A
GSM 106078	131.41	26.26	20
GSM 105989	75.66	8.86	12
GSM 105979	100.97	3.40	3
GSM 105997	>173.90	26.51	N/A
GSM 105972	120.24	9.64	8
GSM 106084	25.7	0	0

‘N/A’ represents cases where the total length data are not precise, and therefore proportions cannot be accurately determined. Specimens from Bed B, Charnwood Forest, UK; housed at BGS.

**Figure 5:**
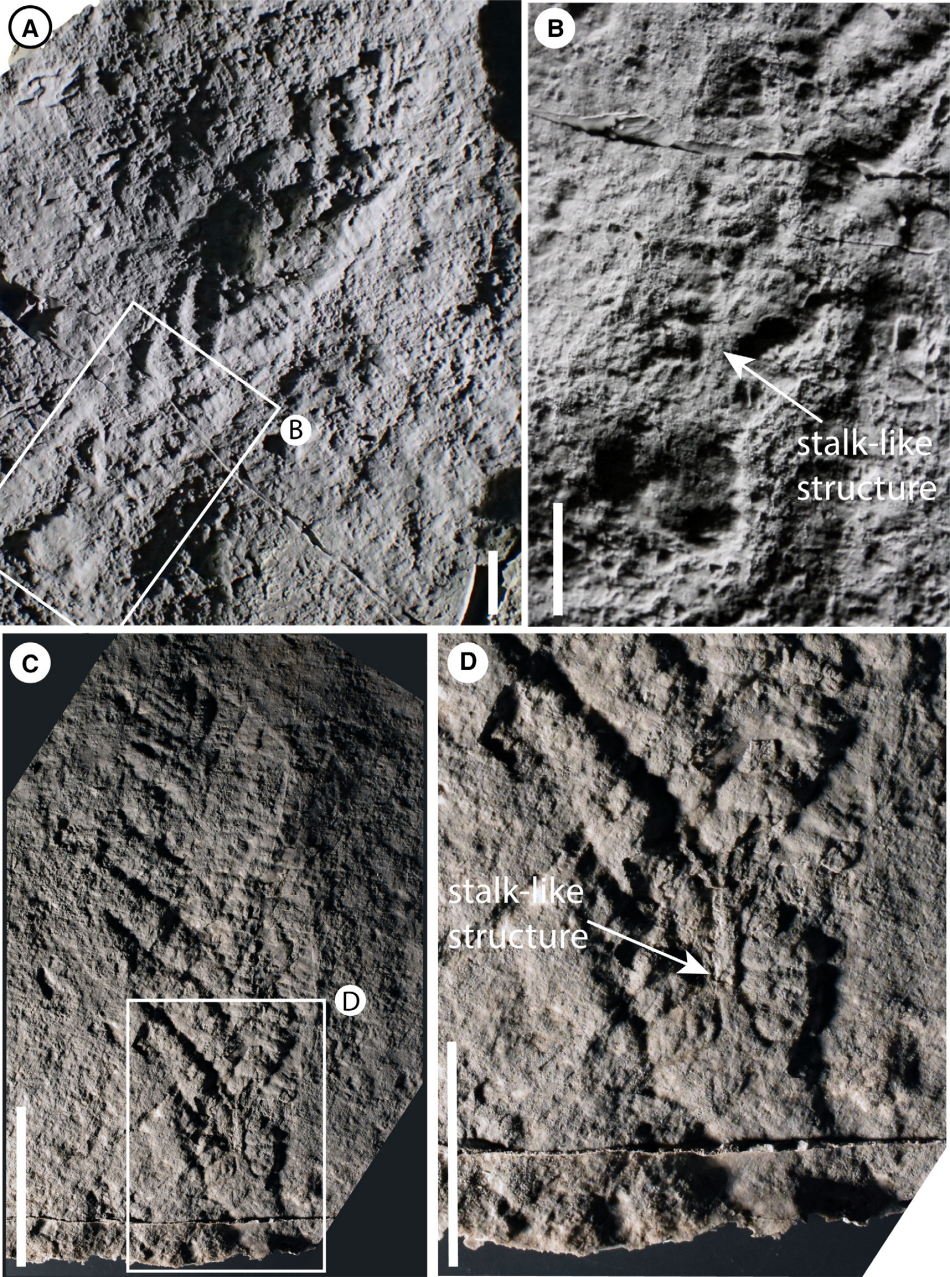
A–B, *Charnia masoni* specimen cast (BGS GSM 105989), Charnwood Forest, UK; B, base of the specimen in A, showing first order branches connecting to a stalk‐like structure (arrowed). C–D, mould of specimen BGS GSM 105997, showing what ostensibly appears to be a stalk‐like structure; D, the stalk‐like region, which appears to represent the effaced remnants of adjacent first order branches. Scale bars represent 10 mm. Colour online.

A stalk‐like structure may be visible near the base of the frond in one specimen (Fig. 5A, B) and appears to connect directly to the holdfast (NB a stalk runs apico‐basally through the frond, and the stem connects the holdfast to the frond, *sensu* Brasier *et al*. 2012). However, similar structures in other specimens appear to be the remains of first order branch boundaries where the branches have been effaced (Fig. 5C, D). Such structures should, therefore, be treated with caution. Where first order branches appear dislocated (Fig. 1F), there does not appear to be any suggestion of a central stalk structure.

A holdfast is not observed in the majority of *Charnia masoni* specimens from Charnwood Forest but where it is observed (16 specimens) it varies from circular to slightly elongate in shape and is generally small (relative to other rangeomorph holdfast structures; e.g. Wilby *et al*. 2011, fig. 2B–C). The possibility remains that it could be deeply buried and therefore not preserved in its entirety on the bedding plane (Burzynski & Narbonne 2015).

### Specimens from Newfoundland

**Figure 6:**
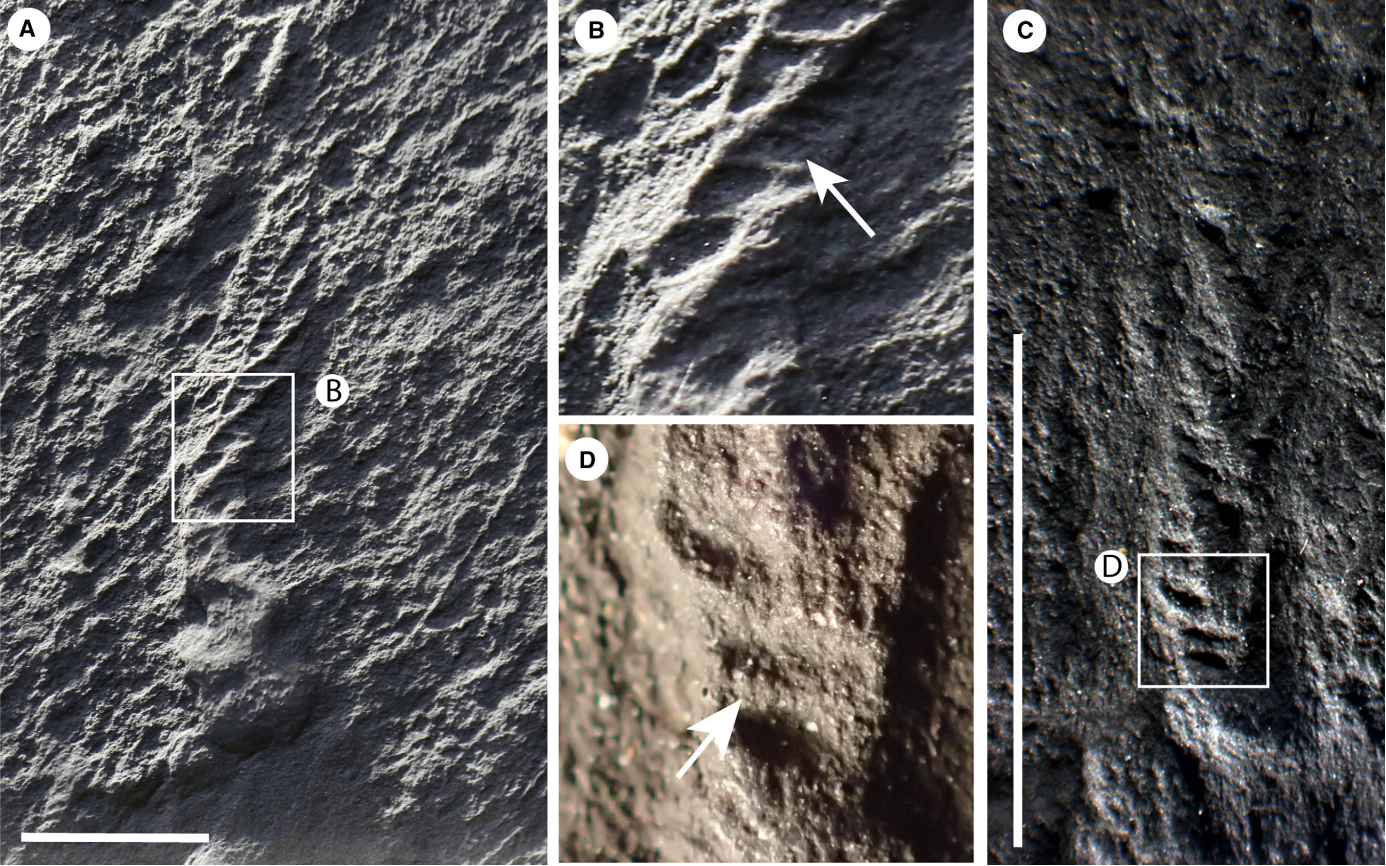
A, *Charnia masoni* (cast) from the MUN surface, Newfoundland, Canada (Liu *et al*. 2016) (CAMSM X.50297.9) showing third order branching, highlighted in B. C–D, the smallest described specimen of *C. masoni* (OUMNH ÁT.429/p) from Pigeon Cove, Newfoundland, Canada (Liu *et al*. 2012) with third order branching highlighted in D. Scale bars represent 10 mm. Colour online.

**Figure 7:**
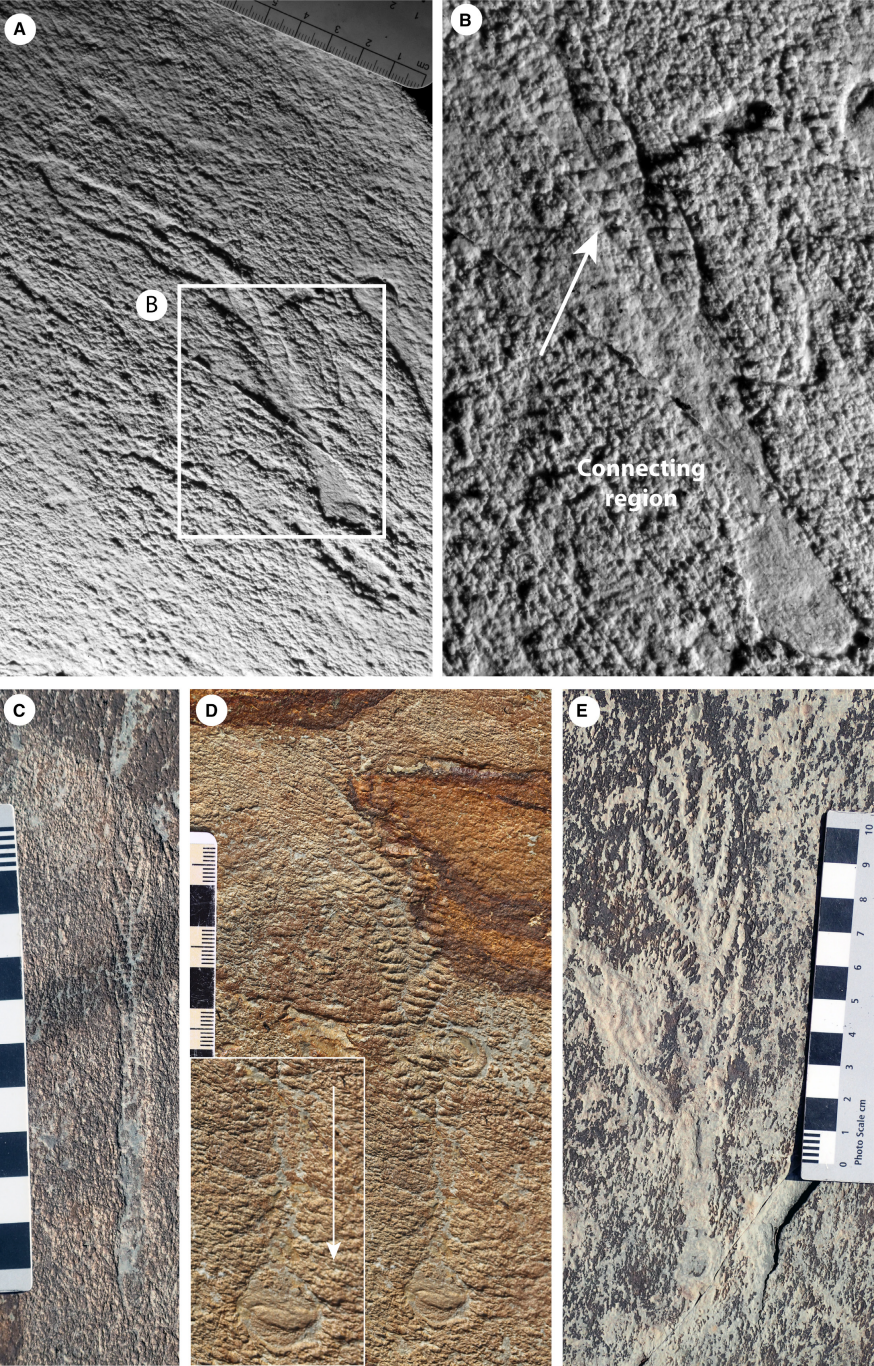
Specimens of *Charnia masoni* from locality LC6, Bonavista Peninsula, Newfoundland, Canada. A–B, silicone mould of a slender (parallel‐sided) specimen (CAMSM X. 50297.10) with what we term the ‘connecting region’, showing sigmoidal first order branching extending much of the way down the specimen, arrowed in B. C, parallel‐sided specimen with a connecting region preserved in positive epirelief (cast of CAMSM X.50297.2). D, specimen with a basal extension in the connecting region (cast of CAMSM X.50297.1); arrow in the inset shows the branch connections to the holdfast. E, Charnwood‐like specimen with first order branches showing ‘connecting region’ typical of parallel‐sided specimens from this surface. Images are retrodeformed, except specimen in C due to lack of available holdfast structures. Main scales in cm. Colour online.

**Figure 8:**
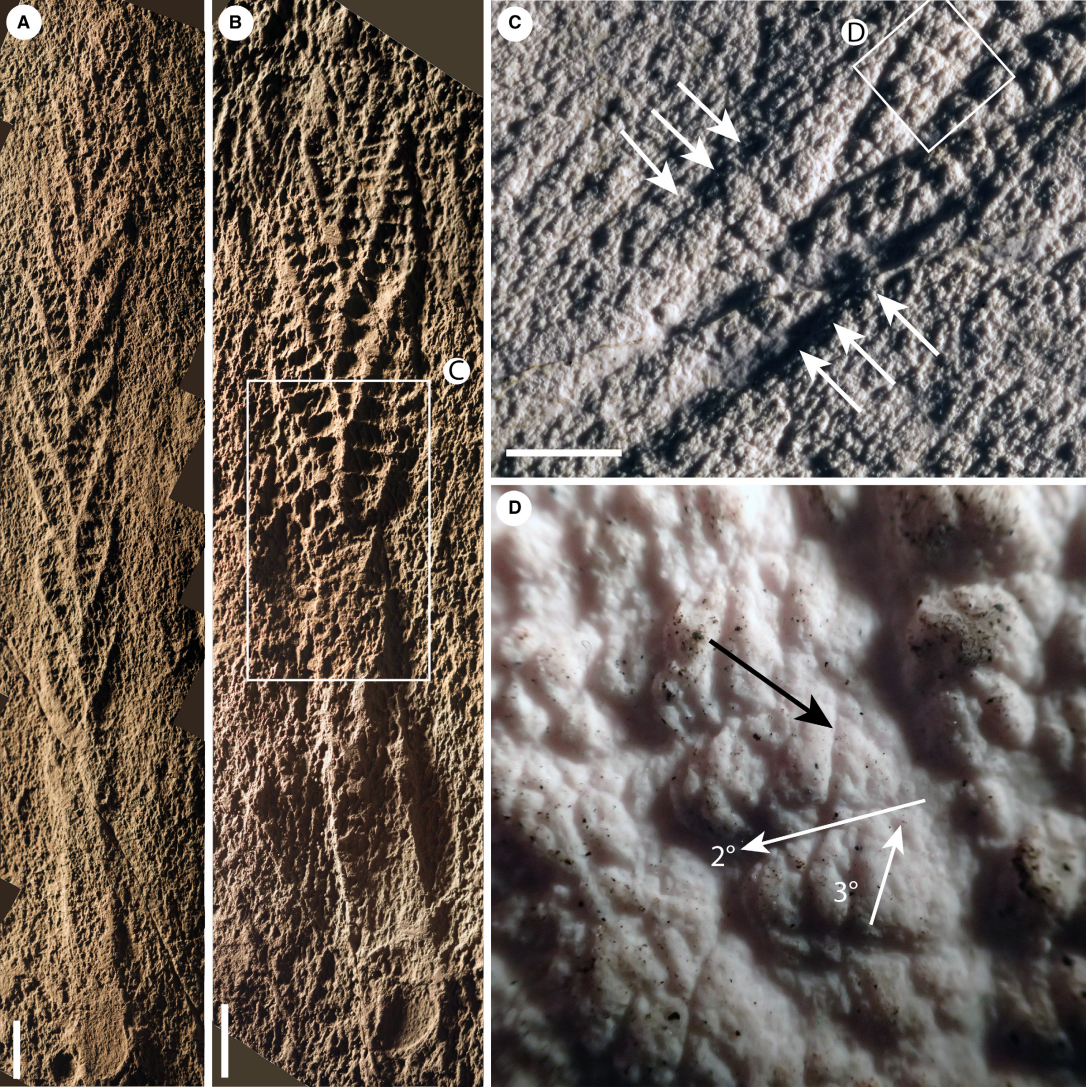
Casts of specimens of *Charnia masoni* from Newfoundland, bed LC6. A, CAMSM X.50297.5. B, CAMSM X.50297.4. C, the basal area of the specimen in B, with second order branches visible (arrowed) on adjacent first order branches running down into the connecting region. D, rotated and furled third order branches, arrowed (black), from the specimen in B (orientation of second and third order branches indicated by white arrows). Images were retrodeformed using the constant area method. Scale bars represent 10 mm. Colour online.

*Charnia masoni* specimens from Newfoundland include small individuals measuring little over 1 cm in length (Liu *et al*. 2012) and possessing three resolvable orders of branching (Fig. 3). Larger specimens may display up to four resolvable orders of branching, with specimens appearing to fall into two distinct morphs that generally show little/no spatial overlap, but which can co‐occur on individual beds. One morph possesses an ovate frond outline, and resembles specimens from Charnwood Forest (e.g. Fig. 6E). The other morph exhibits a slender and strongly parallel‐sided frond (cf. Laflamme *et al*. 2007; Figs 7, 8). Both morphs have a constrained frond form, with the smallest first order branches present at the distal tip of the frond and the longest first order branches present in the middle, with first order branches meeting in the centre of the frond in an alternating arrangement. In the parallel‐sided morph, which is present on at least five distinct surfaces, second order branches appear sigmoidal in shape, where their lateral margins are preserved. Third order branches may be undivided and furled, or rotated and furled (*sensu* Brasier *et al*. 2012; Fig. 8D). Taphonomic constraints prohibit us from drawing conclusions regarding the morphology of the smallest branching orders in the Charnwood‐type morph.

**Figure 9:**
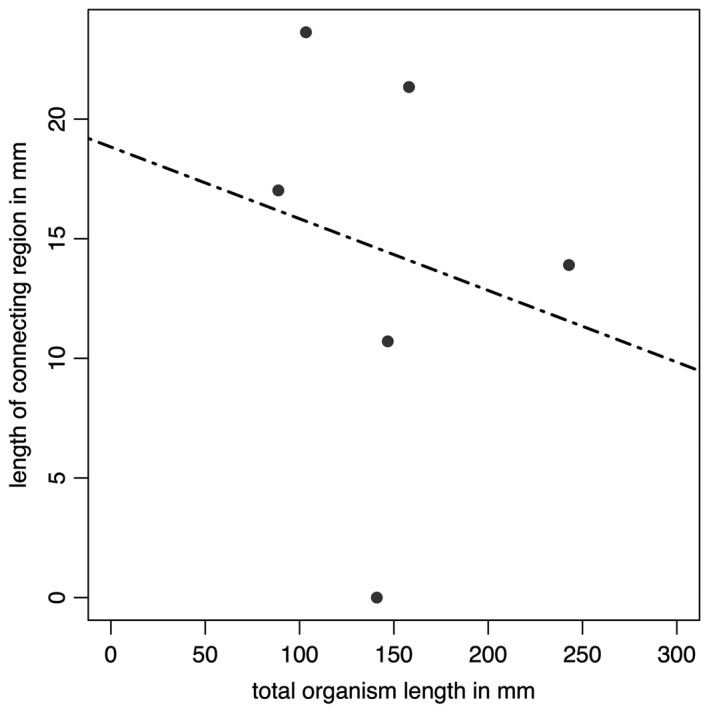
Data from Table 2 plotted in graphical form. The black dashed line represents the best fitting (linear) model (AICc = 59.39492), but this is non‐significant (*p* = 0.7174).

In certain specimens of the parallel‐sided morph from two individual bedding planes in Newfoundland (LC6 and Site 40 of Hofmann et al. 2008), the frond is connected to the holdfast via a long connecting region that is narrower than the frond (Figs 7, 8). On both beds, *Charnia masoni* specimens with this connecting region are considerably more abundant than specimens without (no specimens without the connecting region are documented at Site 40, while only two are documented on LC6, in contrast to *c*. 20 specimens that possess a connecting region). This area is commonly preserved in positive epirelief, in contrast to the negative epirelief preservation of the frond branches (Fig. 7D). It may display first and second order branching at least part way along its length (Figs 7A–C, E; 8), with a bias towards preservation of only one row of first order branches (e.g. Fig. 7B, C). Within this connecting region, effaced first and second order branching is commonly visible (e.g. Figs 7, 8). The length of the connecting region located proximally to the basal‐most expression of distinct first or second order branching is variable within populations (Fig. 9; Table 2) and is not tightly correlated to specimen size. A holdfast is commonly preserved in specimens from Newfoundland, and can exhibit circular to slightly elongate morphologies (Figs 7, 8).

**Table 2:** Comparison of specimen total length and connecting region length in specimens from locality LC6, Newfoundland, Canada

Specimen	Total length (mm)	Connecting region (mm)	Length of connecting region as a proportion of total organism length (%)
X. 50297.11	88.78	15.11	17.02
X. 50297.7	103.4	23.40	23.63
X. 50297.1	140.96	0	N/A
X. 50297.10	146.76	15.72	10.71
X. 50297.4	242.81	33.75	13.9
X. 50297.5	158.01	33.72	21.34

Only specimens where the base of the organism is well preserved were included in our analysis. ‘N/A’ represents cases where the total length data are imprecise, and therefore cannot be used to accurately determine proportions. Images were retrodeformed prior to measurement using the constant area method. Specimens housed at CAMSM.

### Specimens from Russia

**Figure 10:**
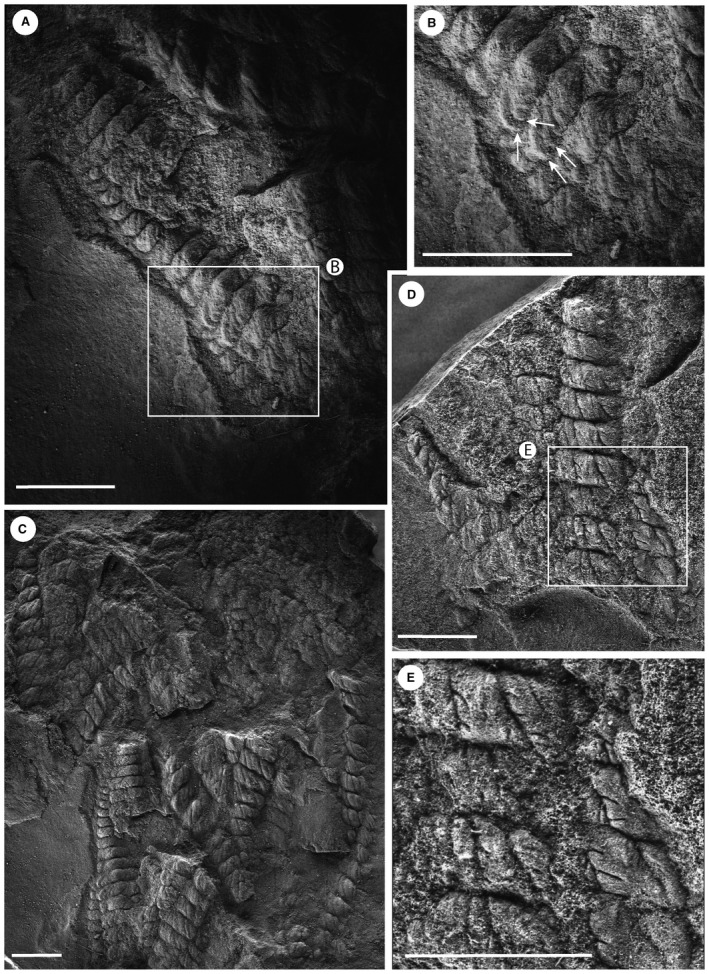
*Charnia masoni* from the Winter Coast of the White Sea, Russia. A–B, PIN 3993‐7023; rotated and furled third order branches evenly spaced at the base of a second order branch but oriented medially at the apex. C, PIN 3993‐7023; clean separations between second order branches, and variation in their width of separation, indicate that the second order branches were probably discrete units each with its own boundary wall (rather than a shared wall with adjacent second order branches). D–E, PIN 3993‐7025; furled, distally inflated fourth order branches (expanded in E) with no further subdivisions visible. Scale bars represent 10 mm.

All examined specimens from the White Sea are incomplete and so no comments about gross form can be made. Four orders of branching were noted in well preserved areas (Fig. 10D, E), and first order branch form appears constrained. First order branches meet along the midline in an alternating fashion, conferring glide symmetry upon the frond. The exposed area in Figure 1D–E (Grazhdankin 2004a, fig. 2B) highlights the tight packing of first order branches. We find no evidence for a central stalk in this exposed area, or in any of the Russian specimens. As with specimens from Newfoundland, second order branches may be rectangular or sigmoidal (furled or displayed; Fig. 10D). Where second order branches are disarticulated (e.g. Fig. 10D), the boundary between these branches appears clean. Third order branches may appear either furled and undivided (Fig. 10A, B), rotated and furled, or displayed and furled (Figs 1D–E, 10E). As with specimens from Newfoundland, the basal margins of third order branches (across one second order branch) are more evenly spaced than the apical margins, which appear to be oriented medially in many cases (e.g. Fig. 10A, B), suggesting that the third order branches attach to a support structure located basally in each second order branch.

## Discussion

**Figure 11:**
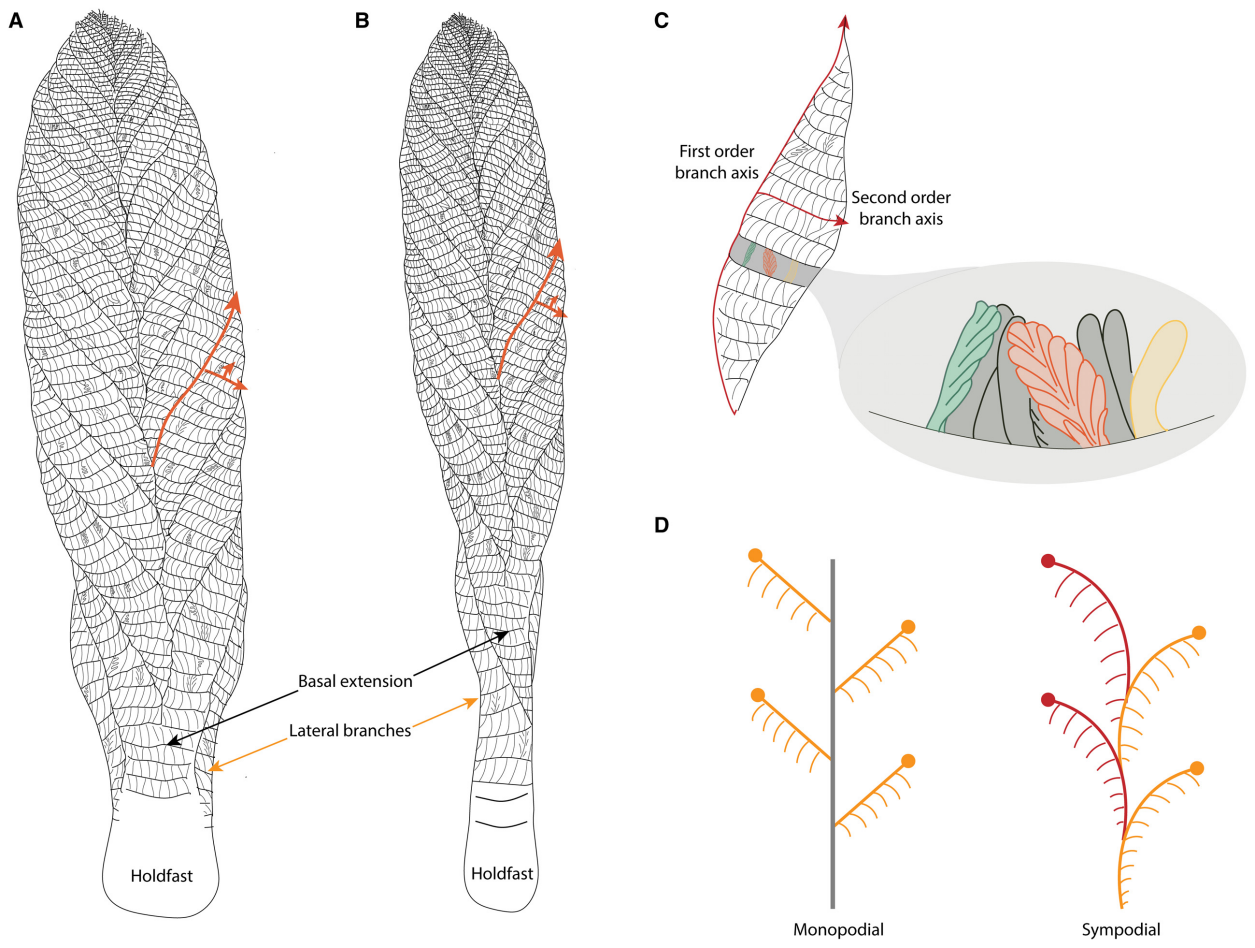
Morphological model of *Charnia masoni*. A–B, Charnwood‐like and parallel‐sided morphotypes of *Charnia masoni*, respectively; orange arrows indicate the orientation of the branch axes up to third order; twisting of central axis is illustrated in B. C, observed variation in third and fourth order branch organization; the orange branch is displayed and unfurled (see Fig. 1C), the green branch is rotated and unfurled (see Fig. 3G) and the yellow branch is undivided and furled (see Fig. 6E); terminology after Brasier *et al*. (2012); red arrows indicate the first order branch axis (oriented apically) and the second order branch axis (oriented laterally). D, monopodial and sympodial central axial arrangements; monopodial growth is characterized by lateral branches emerging from a single central axis, while sympodial growth is characterized by successively stacked lateral branches, without a separate central axial structure (e.g. Berking 2006).

Integration of the information above allows construction of a new morphological model that better reflects the anatomy of *Charnia masoni* (Fig. 11). In the following section, we first discuss the frond and then move basally down the organism to the holdfast.

First order branches in *Charnia masoni* were already known (albeit rarely) to dislocate from each other (Wilby *et al*. 2015), suggesting the presence of only a weak connection between adjacent branches or, alternatively, a stacked arrangement of non‐conjoined branches (bound together only at the central axis, or alternatively attached to an axis independent of each other). Evidence indicating that the basal margin of one first order branch could overlie the apical margin of the previous first order branch (Grazhdankin 2004a, fig. 2D; Laflamme *et al*. 2007) perhaps supports the latter hypothesis. We do not find evidence for a marginal rim (*sensu* Narbonne *et al*. 2009), or any other connective structure inferred to surround first order branches. *Charnia masoni* possesses three further orders of branch subdivision (totalling four orders of branching). It is not currently possible to determine whether the observation that only three branching orders are visible in the smallest, presumed youngest, specimens results from ontogenetic or taphonomic processes.

First order branches are sigmoidal in shape and are constructed of second order branches that are rectangular to sigmoidal. Variation in second order branch morphology is the result of the degree of physical rotation each branch has undergone, with fully exposed branches appearing sigmoidal (e.g. Fig. 6; see also Laflamme *et al*. 2007), whereas rectangular second order branches appear to have been furled. Second order branches probably possessed their own boundary walls and so it is unlikely that they were joined to each other in life along their entire medial‐distal axis; they were connected only at their medial margin. We therefore term this medial margin the first‐order branch axis (Fig. 11C).

We see no evidence to suggest that first or second order branches in *Charnia masoni* could exhibit a displayed rangeomorph branching architecture in any examined specimens, consistent with previous suggestions of single‐sided ‘Charniid’ branching at these branch orders (Narbonne *et al*. 2009; see also thin section data in Grazhdankin 2004a, fig. 2d.)

While the majority of third order branches appear to conform to the typical furled, rotated or undivided, rotated pattern that defines the genus (e.g. Brasier *et al*. 2012; Wilby *et al*. 2015) individual branches at these higher orders may be furled and displayed, while some are unfurled and displayed (Fig. 1C). Given the apical orientation of displayed third order branches in specimens from Charnwood Forest, as well as the apical margins of third order branches in specimens from Russia being oriented medially (thus suggesting they were not bound at this margin), third order branches are interpreted as branching apically from their host second order branch along a second order branch axis (Fig. 11C). Third order branches also exhibit moderate inflation (*sensu* Brasier *et al*. 2012). Given the rotational variation we observe in fourth order branching, we consider it unlikely that third order branches were conjoined.

Fourth order branches have never been observed to show further hierarchical subdivision. We acknowledge that taphonomic constraints may preclude visualization of further branch orders but note that space constraints do not appear to limit the number of orders visible (e.g. Fig. 10E). Fourth order branches typically appear furled and may exhibit moderate (Fig. 1C) or medial (Fig. 10E) inflation. This is unlike the apparently conserved proximal inflation inferred for first order branches but similar to the moderate–medial inflation inferred for second order branches (Brasier *et al*. 2012).

These observations help to resolve the long‐standing question regarding whether rotated (*sensu* Brasier *et al*. 2012) or ‘charniid’ branches (*sensu* Narbonne *et al*. 2009) have one or two rows. These specimens (from Charnwood, UK and the White Sea, Russia) demonstrate that rotated branches could be two‐sided at higher branch orders, with one side rotated out of the plane of preservation (Fig. 11C). The potential for (at least third order) rotated branches to appear displayed (Fig. 10D, E), and furled branches to appear unfurled (Fig. 1C), suggests branching characters at higher (third and fourth) orders are not taxonomically conserved (see Kenchington & Wilby 2017). The rotation of these branches supports the notion that at least fourth order branches, and perhaps third order branches in *Charnia masoni*, were not conjoined, but free to move and rotate in the axial plane (cf. Wilby *et al*. 2015).

Branching architecture has significant bearing on the debate surrounding whether *Charnia masoni* had distinct front–back differentiation (see also Grazhdankin 2004a). We have been unable to corroborate the identification of two different faces to *C. masoni* in the *c*. 70 specimens directly studied here, and therefore infer that both sides of the organism probably possessed the same morphology (see also a Charnwood specimen inferred to be twisted (*sensu* Wilby *et al*. 2015) but displaying the same morphology above and below the twist: Fig. 1F). The apparent absence of third and fourth order branching in some specimens from the White Sea (Grazhdankin 2004a, fig. 2A) may then represent a taphonomic artefact. The considerable morphological variation in third and fourth order branches (as opposed to first and second order) may suggest that these finer orders of branching played a greater role in nutrient acquisition, as they were free to rotate around their axis. However, this greater flexibility could also simply be a function of their small size and not have been of functional significance. The lack of evidence for rangiid style branching in the first and second order branches may further suggest that *C. masoni* is not self‐similar at every branch order (e.g. Narbonne 2004), although additional evidence is required to confirm or refute this. If this suggestion is borne out, this would undermine the current definition of Rangeomorpha, which requires orders of branching that are identical to ‘at least three orders’ (Erwin *et al*. 2011).

The lateral branches (Fig. 2) are morphologically distinct in terms of their unique attachment point to the holdfast, perhaps indicating a greater level of axial complexity to *Charnia masoni* than has previously been inferred (e.g. Hoyal Cuthill & Conway Morris 2014, though see Dunn *et al*. 2018a *a*). The next most proximal pair of first order branches may also be morphologically distinct, in some cases extending between the two most proximal first order branches to form an area previously termed the ‘stem’ (e.g. Dunn *et al*. 2018a *a*; Fig. 1B). However, because this area, where present, comprises two individual first order branches rather than a central fused region, we term this area the ‘basal extension’ (Figs 1A–B, 11). The basal extension displays some similarity to the proximal section of the subdivided ‘axial stalk’ (a stem as defined by Brasier *et al*. 2012) described in *Rangea schneiderhoehni* (Vickers‐Rich *et al*. 2013). However, in *R. schneiderhoehni* this area is considered a single structure (i.e. not constructed of abutting first order branches). The basal extension is also distinct from the ‘naked’ stems of other rangeomorphs (e.g. Laflamme *et al*. 2012).

The parallel‐sided morph of *Charnia masoni* from Newfoundland possesses a connecting region (Figs 7, 8), although exact structural reconstruction of this region is hampered by variable quality of preservation near the base of the frond (resulting in often gradational boundaries between the branched area and ‘naked’ connecting region). This gradational zone, with what appear to be first order branches continuing down the ‘connecting region’ in many specimens (e.g. Figs 7A–B, 8A), suggests that this area does not represent a sheath structure (Narbonne *et al*. 2009; though see Brasier *et al*. 2013). This structure could alternatively be interpreted as an artefact of dragging upon felling. However, the presence of clear (if weakly) demarked first and second order branches that are both aligned with and fit the size profile of other branches in the frond, renders this interpretation unlikely. Laflamme *et al*. (2007) documented the parallel‐sided morph from Lower Mistaken Point on the Avalon Peninsula, but do not describe any form of connecting region, with branches connecting directly to the holdfast (their fig. 6I–J), providing further support that this area may not be a ‘stem’. Taken together with the variability in presence and appearance of branches in the connecting region in bedding plane populations of specimens in Newfoundland, the connecting region is likely to represent an artefact of specimen twisting upon felling and burial. Twisting would not necessarily affect branch preservation in more apical regions but could result in the apparent absence or poor preservational fidelity of branches closer to the base of the frond.

The base of the *Charnia masoni* frond thus appears to reflect an area with considerable morphological variation, perhaps resulting from taphonomic, environmental, and/or biological factors. The proportional length of this region is variable even across specimens of a similar size from the same bedding plane (Fig. 4; Table 1). Some of this intra‐specific variation may suggest a hitherto unrecognized plastic element to *C. masoni* growth and morphology, and a potential capacity to respond to local environmental factors (e.g. neighbour competition or nutrient availability) by differential growth (cf. Hoyal Cuthill & Conway Morris 2017; Kenchington & Wilby 2017).

None of the specimens examined show evidence for an internal stalk running along the length of the organism, such as that seen in other rangeomorphs (e.g. *Avalofractus abaculus*, Narbonne *et al*. 2009, or *Rangea schneiderhoehni*, Vickers‐Rich *et al*. 2013; Sharp *et al*. 2017). Stalk‐like structures observed in our investigations are interpreted as the effaced remains of first order branch margins (Fig. 2C, D). Indeed, space constraints (highlighted by Grazhdankin 2004a) mean that the presence of such a stalk in *Charnia masoni* is unlikely. An alternative scenario involves the central axis in *C. masoni* being constructed by successively stacked lateral branches (schematically represented in Fig. 11D), conferring a sympodially organized central axis (as opposed to the monopodial arrangement present in *Avalofractus* or *Rangea*). We note here the distinctive nature of the basal‐most branches in *C. masoni*, which differentiate directly from the holdfast (Dunn *et al*. 2018a *a*). However, we acknowledge that it currently remains difficult to differentiate between these two possible axial arrangements based on the available evidence.

Previous taxonomic schemes for rangeomorphs have placed emphasis on an internal stalk (Laflamme & Narbonne 2008; Brasier *et al*. 2012) and whether it is exposed or concealed. Narbonne *et al*. (2009) illustrated a structure they interpreted as an internal stalk in a *Charnia*‐like frond. However, this structure could alternatively be explained by sedimentary or taphonomic processes (Grazhdankin & Seilacher 2005; Brasier *et al*. 2013) and, given the very small number of such known examples, we do not consider it a compelling morphological feature. Stalks (as opposed to stems) are assumed but not demonstrated to be present in several other rangeomorphs including the uniterminal *Beothukis mistakensis* and *Beothukis plumosa*, or the biterminal rangeomorph genus *Fractofusus*. Some extant frondose organisms (e.g. hydrozoan cnidarians) are known to display variation in axial arrangement within a clade (e.g. Berking 2006) and so the idea that, even if they are a monophyletic group, all rangeomorphs must share similar axial arrangements may be erroneous.

The morphology of the holdfasts in *Charnia masoni* can vary markedly between different specimens (Grazhdankin *et al*. 2008, fig. 2A; Wilby *et al*. 2015, fig. 4), ranging from circular to diamond in shape. This variation could represent either true biological or taphonomic variation (Burzynski *et al*. 2017), or a combination of the two. The most parsimonious scenario is that differing depths of holdfast burial account for the majority of observed variation in our studied populations.

The redescription of *Charnia masoni* allows construction of a new model for its *in vivo* anatomy (Fig. 11). The organism was attached to the sediment by a bulbous holdfast and was constructed of a series of stacked first order branches arranged in two rows, which may have been derived successively from a sympodial central axis, or from a cryptic monopodial axis. Each first order branch had an apical axis from which a series of second order branches emerged laterally. Third order branches were attached to the second order branch axes and were oriented apically. Variation in both original anatomy and preservation near the base of the organism results in the variable presence or absence of both a basal extension, and the lateral branches.

## Conclusions

Evaluation of the morphology of *Charnia masoni* from three late Ediacaran assemblages (Charnwood Forest, Newfoundland, and the White Sea) enables assembly of an emended model of morphology for this organism, demonstrating greater levels of intraspecific variation than have previously been documented. *C. masoni* specimens from the different localities are comparable in morphology but show features that cannot easily be reconciled with previous rangeomorph taxonomic regimes, and potentially fall outside the current definition of Rangeomorpha. Our study reveals that certain characters previously proposed as taxonomically informative, such as the displayed/undisplayed, furled/unfurled nature of branches, are fallible at higher branch orders. We provide an emended diagnosis of *Charnia masoni* to take account of the novel features and variation described herein (see below).

A more detailed understanding of anatomy must necessarily precede phylogenetic interpretation, since organisms must be interpreted as the sum of all their parts. Our novel interpretation of anatomy in *Charnia masoni*, an organism that is among the most widely studied of the Ediacaran macrobiota, illustrates the potential for obtaining significant amounts of new information from global‐scale, population‐wide studies of well preserved Ediacaran specimens.

## Systematic palaeontology

#### Genus CHARNIA Ford 1958

###### Emended diagnosis

Frond uniterminal, comprising two rows of non‐conjoined first order branches arranged alternately along a central axis, presenting as a zig‐zag medial suture. First order branches typically show proximal inflation, whereas (non‐conjoined) second‐order units show moderate‐to‐medial inflation. All first to fourth order branches are aligned in subparallel series. Second order branches are oriented basally, whereas first and third order branches are oriented apically. First order branches comprise rangeomorph elements that are rotated and undisplayed, while second order branches are comprised of rangeomorph elements that may be rotated and either furled or unfurled. There is variation in the presentation of third and fourth order rangeomorph branch elements, which can be displayed and unfurled, displayed and furled, undisplayed and furled, or undivided. A basal disc is present in some specimens.

###### Type species

*Charnia masoni* Ford, 1958.

#### *Charnia masoni* Ford, 1958

v* 1958: *Charnia masoni* Ford, p. 212, pl. 13, fig. 1.
? 1959: *Charnia* sp.; Glaessner, p. 1472, text‐fig. 1b.
? 1959: *Rangea?*; Glaessner, *in* Glaessner & Daily, p. 387, pl 46, fig. 2.
1961: *Charnia* sp.; Glaessner, p. 75, text‐fig.
1962: *Charnia* sp.; Glaessner, pp 484–485, pl. 1, fig. 4 (*non* fig. 5).
1962: *Charnia masoni*; Ford, fig. 4 (*non* fig. 5).
1966: *Rangea grandis*; Glaessner & Wade, p. 616, pl. 100, fig. 5.
1972a: *Rangea sibirica*; Sokolov, pl. I, fig. 3.
1972b: *Rangea sibirica*; Sokolov, p. 50.
1973: *Glaessnerina grandis*; Germs, p. 5, fig. 1D.
1976: *Charnia* ex gr. *masoni*; Sokolov, p. 141.
1977: *Charnia* ex gr. *masoni*; Sokolov, p. 441.
1978: *Charnia masoni*; Fedonkin, fig. 3 (9).
1979: *Charnia masoni*; Glaessner, fig. 12 (3).
1979: *Glaessnerina sibirica*; Glaessner, fig. 12 (1).
1981a: *Charnia masoni*; Fedonkin, p. 66, pl. 3, figs 5, 6; pl. 29, fig. 1.
1981a: *Zolotytsia biserialis*; Fedonkin, p. 67–68, pl. 3, fig. 7.
1981b: *Charnia masoni*; Fedonkin, p. 100.
1981: *Charnia masoni*; Sokolov & Brekhovskikh, p. 3.
1981: *Glaessnerina grandis*; Glaessner & Walter, fig. 6.11 (C).
1983a: *Charnia masoni*; Fedonkin, fig. 37.
1983b: *Charnia masoni*; Fedonkin, pl. 1, fig. 1.
1983: *Charnia masoni*; Sokolov & Fedonkin, p. 13, fig. 9.
1984: *Charnia masoni*; Sokolov, p. 6, fig. 1.
1984: *Charnia masoni*; Glaessner, fig. 2.21 (A).
1984: *Glaessnerina sibirica*; Glaessner, fig. 2.21 (D).
1984: *Glaessnerina grandis*; Glaessner, fig. 2.21 (C).
1984: *Charnia masoni*; Sokolov & Fedonkin, fig. 3 (f).
1984: *Charnia* cf. *C. masoni*; Glaessner, fig. 2.21 (B).
1985: *Charnia masoni*; Fedonkin, p. 99, pl. 12, fig. 4; pl. 13, figs 2–4.
1985: *Charnia* cf. *C. masoni*; Jenkins, fig. 7 (C).
1985: *Charnia masoni*; Jenkins, fig. 7 (B).
1987: *Charnia masoni*; Fedonkin, pl. 15.
1987: *Glaessnerina grandis*; Preiss, p. 310, fig. E.
1990: *Charnia masoni*; Fedonkin, fig. 1 (D).
1992: *Charnia masoni*; Fedonkin, fig. 28–30.
1992: *Charnia masoni*; Runnegar & Fedonkin, fig. 7.5.5 (A), fig. 7.5.10 (A).
1994: *Charnia masoni*; Fedonkin, fig. 2 (A, B).
v* 1995: *Charnia grandis*; Boynton & Ford, p. 168, fig. 1.
1996: *Glaessnerina grandis*; Jenkins, p. 35, fig. 4.1.
v 1997: *Charnia masoni*; Grazhdankin & Bronnikov, p. 794, fig. 2 (a, d).
? 1998: *Charnia masoni*; Nedin & Jenkins, p. 315, fig. 1.
1999: *Charnia grandis*; Ford, p. 231, fig. 3.
v 2000: *Charnia*; Martin *et al*., fig. 4 (A).
v 2004a, 2004b: *Charnia*; Grazhdankin, p. 207, fig. 2.
2005: *Charnia masoni*; Narbonne *et al*., p. 28, pl. 1L.
v 2005: *Charnia*; Grazhdankin *et al*., fig. 3 (d).
v 2007: *Charnia masoni*; Laflamme *et al*., p. 243, fig. 4A–J.
v 2007: *Charnia* sp.; Fedonkin *et al*., p. 128, fig. 232 (*partim*).
v 2007: *Charnia* cf. *masoni*; Fedonkin *et al*., p. 145, fig. 276 (*partim*).
v 2007: *Charnia* cf. *masoni*; Fedonkin *et al*., p. 160, 165, figs 304, 314 (*partim*).
v 2007: *Charnia masoni*; Fedonkin *et al*., p. 186, fig. 354.
2008: *Charnia masoni*; Hofmann *et al*., p. 17 (*partim*), fig. 13.1.
v 2008: *Charnia grandis*; Hofmann *et al*., p. 18, fig. 14.
v 2008: *Charnia masoni*; Grazhdankin *et al*., p. 804, fig. 2A.
v 2009: *Charnia masoni*; Bamforth & Narbonne, p. 907, fig. 7.5.
v 2011: *Charnia masoni*; Wilby *et al*., pp 656–657 (*partim*), figs 2A, 3A.
v 2011: *Charnia masoni*; Grazhdankin, fig. 3 (a–d).
v 2012: *Charnia masoni*; Liu *et al*., p. 397, figs 4B, 5A.
v. 2013: *Charnia* aff. *masoni*; Liu *et al*., p. 24, fig. 1D.
v 2013: *Charnia masoni*; Liu *et al*., p. 24, fig. 2A–D.
2013: *Charnia* sp.; Gehling & Droser, p. 449, fig. 2Q.
v 2014: *Charnia masoni*; Grazhdankin, p. 271 fig. 2.3.
v 2015: *Charnia masoni*; Wilby *et al*., p. 20, fig. 2.1,3,6, fig. 2.2,4, fig. 2,5.
v 2015: Incomplete frond; Wilby *et al*., p. 20, fig. 2.8.
v 2015: *Charnia masoni*; Liu *et al*., p. 1361, fig. 2D.
v 2016: *Charnia masoni*; Liu *et al*., p. 5 *(partim),* fig. 3D.
v 2017: *Charnia masoni*; Antcliffe *et al*., p. 27, fig. 4E.
v 2018a*a*: *Charnia masoni*; Dunn *et al*., p. 5, fig. 1E, p. 7, fig. 3.

###### Diagnosis

As for the genus.

###### Remarks

We do not consider the described variation between specimens of *Charnia masoni* from Charnwood, Russia and Newfoundland to be taxonomically significant. Following recent taxonomic discussions on rangeomorphs, we consider all studied specimens to at least belong within the same genus (cf. Liu *et al*. 2016; Kenchington & Wilby 2017). Determination of whether the specimens represent morphs of the same species, or separate species, is more challenging. Where there is variation in multiple continuous characters within Ediacaran taxa, it has been proposed that this would be sufficient to indicate species level differences (Liu *et al*. 2016), depending on the nature and extent of this variation (Kenchington & Wilby 2017). However, when considering morphs from different localities, it can be extremely difficult to distinguish between taxonomic and intraspecific variation (Kenchington & Wilby 2017). Although both parallel‐sided (Newfoundland) and ovate (Charnwood, White Sea) morphs of *C. masoni* may be present on individual surfaces (e.g. Fig. 7, from Bed LC6), such occurrences are rare and there is typically one numerically dominant morph.

If further variation (categorical or continuous *sensu* Kenchington & Wilby 2017) is described in these morphs, we would consider it appropriate to reassess these conclusions. Indeed, if variation in discrete characters is identified, then it may be appropriate to erect a new genus.
